# Supplementary material for: Content-rich biological network constructed by mining PubMed abstracts
Source: BMC Bioinformatics. 2004 Oct 8;5:147. doi: 10.1186/1471-2105-5-147 (PMC528731; doi:10.1186/1471-2105-5-147)
Supplement: Additional File 2 — The original results of the above study (non-essential files are deleted to keep the file size under the limit set by BMC bioinformatics). [file 1471-2105-5-147-S2.bz2 › chilibotAdditionalFile2/dip05/386ID10318834E9856/html/EPO_EPOR.html]

 


 **EPO** and **EPOR** 
  
Found 716 abstracts in PubMed, retrieved 05.  
 

 What does Google say? 
 PDF only 
| .edu only 

---

**Interactive relationship** (e.g. stimulation, inhibition, etc)

**Neutral relationship**- Erythropoietin  [ **EPO** ]   **EPO**  regulates mammalian erythropoiesis by binding to its transmembrane receptor  **EPOR** .  Ref: 12878211 Biochem Biophys Res Commun, 2003
- These findings indicate the expression of multiple  **EPOR**  isoforms in human cancer cells that may modulate the cellular effects of recombinant human  **EPO**  or  **EPO**   **EPOR**  antagonists.  Ref: 12878211 Biochem Biophys Res Commun, 2003
- Signals provided by the  **Epo**  receptor  **EpoR**  are required for erythroid development beyond the CFUe stage, and are propagated via the  **EpoR**  tethered Janus kinase, JAK2.  Ref: 12869513 BloodBlood, 2003
- Presently, the activities of a PY null  **EpoR**  HM form in primary progenitor cells from knock in mice were further assessed using optimized  **Epo**  dose dependent proliferation, survival and differentiation assays.  Ref: 12869513 BloodBlood, 2003

**Non-interactive relationship** (e.g. studied together, co-existance, homology, etc.)

- It has previously been demonstrated that mutations in the 232 WSAWS 236 motif in the erythropoietin  [ **EPO** ]  receptor  **EPOR**  can result in strongly inhibited surface expression, due to defective intracellular transport Hilton, D.  Ref: 12859190 Biochemistry, 2003
- As compared to the wt  **EpoR** ,  **EpoR**  HM activity was compromised several fold in each context when  **Epo**  was limited to physiological concentrations.  Ref: 12869513 BloodBlood, 2003
- Furthermore, administration of  **EPO**   **EPOR**  antagonists delayed the growth of uterine, ovarian, and mammary carcinoma cells in experimental animal models.  Ref: 12878211 Biochem Biophys Res Commun, 2003
